# High frequency of cutaneous manifestations including vitiligo and alopecia areata in a prospective cohort of patients with chronic graft-vs-host disease

**DOI:** 10.3325/cmj.2016.57.229

**Published:** 2016-06

**Authors:** Romana Čeović, Lana Desnica, Dražen Pulanić, Ranka Serventi Seiwerth, Ivana Ilić, Magdalena Grce, Marinka Mravak Stipetić, Tajana Klepac Pulanić, Ervina Bilić, Ernest Bilić, Milan Milošević, Radovan Vrhovac, Damir Nemet, Steven Z Pavletic

**Affiliations:** 1 Department of Dermatology and Venereology, University Hospital Centre Zagreb, Zagreb, Croatia; 2 University of Zagreb School of Medicine, Zagreb, Croatia; 3 Division of Hematology, Department of Internal Medicine, University Hospital Centre Zagreb, Zagreb, Croatia; 4 Faculty of Medicine Osijek, J.J. Strossmayer University of Osijek, Osijek, Croatia; 5 Department of Pathology and Cytology, University Hospital Centre Zagreb, Zagreb, Croatia; 6 Division of Molecular Medicine, Ruđer Bošković Institute, Zagreb, Croatia; 7 University of Zagreb, School of Dental Medicine, Zagreb, Croatia; 8 University Dental Clinic, Clinical Department of Oral Medicine, University Hospital Centre Zagreb, Zagreb, Croatia; 9 Department of Gynecology, Community Health Center Zagreb East, Zagreb, Croatia; 10 Department of Neurology, University Hospital Centre Zagreb, Zagreb, Croatia; 11 Department of Pediatric Hematology and Oncology, University Hospital Centre Zagreb, Zagreb, Croatia; 12 University of Zagreb, School of Medicine, Andrija Štampar School of Public Health, Zagreb, Croatia; 13 Experimental Transplantation & Immunology Branch, Center for Cancer Research, National Cancer Institute, National Institutes of Health, Bethesda, MD, USA

## Abstract

**Aim:**

To determine the frequency and the characteristics of cutaneous manifestations, especially vitiligo and alopecia areata, in patients with chronic graft-vs-host disease (cGVHD).

**Methods:**

50 patients with cGVHD were prospectively enrolled in the observational study protocol and evaluated by an experienced dermatologist. The evaluation was focused on the clinical spectrum of skin and adnexal involvement, and the cutaneous GVHD score was determined according to National Institutes of Health (NIH) Consensus criteria. The presence of vitiligo, alopecia, xerosis, nail changes, and dyspigmentation was also assessed.

**Results:**

Out of 50 cGVHD patients, 28 (56%) had skin involvement, and 27 of them (96%) had hypo and/or hyperpigmentations. 11 patients (39%) had a mild cutaneous NIH cGVHD score, 22% moderate, and 39% severe. 15 (30%) patients had nail changes and 10 (20%) had vitiligo or alopecia areata. Univariate analysis showed that patients with vitiligo/alopecia areata received more lines of prior systemic immunosuppressive therapy (*P* = 0.043), had lower Karnofsky performance status (*P* = 0.028), and had a higher B-cell number (*P* = 0.005), platelet count (*P* = 0.022), and total protein (*P* = 0.024). Vitiligo and alopecia areata were associated with higher NIH skin score (*P* = 0.001), higher intensity of immunosuppressive treatment (*P* = 0.020), and total body irradiation conditioning (*P* = 0.040). Multivariate regression model showed that patients with higher NIH skin scoring were 3.67 times more likely to have alopecia and/or vitiligo (odds ratio 3.67; 95% confidence interval 1.26-10.73), controlled for all other factors in the model (age at study entry, number of B-cells, platelet count, and global NIH score).

**Conclusion:**

These data indicate that vitiligo and alopecia areata occur more frequently in cGVHD than previously reported.

Graft-vs-host disease (GVHD) is a complex multi-organ disease that occurs in patients after allogeneic hematopoietic stem cell transplantation (allo-HSCT) (1). Based on the consensus recommendations of the GVHD working group of the US National Institutes of Health (NIH), GVHD may be divided into classic acute GVHD, persistent, recurrent or delayed-onset acute GVHD, and classic chronic GVHD and overlap chronic GVHD (2).

At the time of the initial chronic GVHD (cGVHD) diagnosis, most commonly involved organ system is the skin, in approximately 75% of patients, followed by, in decreasing frequency, the oral mucosa, liver, and eye (3,4). Chronic GVHD may affect various skin compartments –the epidermis, dermis, and subcutis (1). Diagnostic skin manifestations of cGVHD are poikiloderma, lichen planus-like eruptions, lichen-sclerosus-like lesions, morphea-like sclerosis, and deep sclerosis/fasciitis and therefore there is no need for a biopsy to establish the diagnosis (2). Although these changes are usually readily evaluated by an experienced dermatologist, differential diagnoses may be necessary. If there is uncertainty, a biopsy should be performed (1). Consensus on performing skin biopsies in suspected cGVHD was published by Hillen et al (5). Among less typical manifestations of cGVHD are ichthyosiform presentations, depigmentation, follicular erythematous papules resembling keratosis pillars, psoriasisform plaques, and annular erythematous lesions (1). Sometimes, there is involvement of the scalp, which may present as scarring or nonscarring alopecia. Various nail changes, including thinning of the nail plate, nail dystrophy with longitudinal ridging, or complete loss of the nail are often found among patients with cGVHD.

Chronic GVHD is known to be associated with dysregulation of the host immune system and could act as a triggering factor for the appearance of autoimmune diseases such as vitiligo and alopecia areata (6). However, reports on vitiligo and alopecia areata in chronic GVHD studies are exceptionally rare, and mostly are case studies. Therefore, the aim of this study was to determine the frequency and characteristics of dermatological manifestations, with an emphasis on vitiligo and alopecia areata, in a well characterized prospectively incepted study cohort of Croatian patients with cGVHD.

## Patients and methods

### Study cohort

This study is a part of the protocol entitled “Clinical and Biological Factors Determining Severity and Activity of Chronic Graft-Versus-Host Disease After Allogeneic Hematopoietic Stem Cell Transplantation.” Patients were prospectively enrolled into the study at the University Hospital Center Zagreb from June 2013 to December 2015 and evaluated for cGVHD by a multidisciplinary team of subspecialists in hematology, dermatology, dentistry, ophthalmology, physical therapy, gynecology, neurology, and other specialist if needed. 45 adults and 5 pediatric patients were evaluated and diagnosed with cGVHD according to the 2005 NIH Consensus criteria (2). Laboratory workup was done and quality of life questionnaires were filled out at the study entry. All patients signed informed consent approved by the Ethical Committee of the University Hospital Center Zagreb.

### Dermatologic evaluation

Dermatologic evaluation was focused on determining the cutaneous GVHD score according to NIH criteria, assessment of other objective clinical parameters including the presence of vitiligo, xerosis, alopecia, nail changes, and dyspigmentation. During the evaluation of skin changes, the Cutaneous GVHD Assessment Worksheet was completed, and the Total Dermatology Score was tallied (7). Skin involvement was determined by the scoring system according to the 2005 National Institutes of Health (NIH) Consensus Criteria (2). Skin disease severity was divided into levels ranging from 0 to 3 based on the features and the extent of involvement. Extent (% body surface area, BSA) of involvement was estimated for lichenoid, sclerotic, fascial disease, and/or regions with bullae, erosions, or ulcers. A score of 0-3 (none, mild, moderate, severe) was given, which directly corresponds to BSA affected. The NIH scores were categorized as mild (1-2 organs with a score 1), moderate (≥3 organs with a score 1, any organ with a score of 2, or a lung with a score of 1), and severe (any organ with a score of 3 or a lung with a score ≥2). The scoring was performed by an experienced dermatologist. Skin biopsy was performed in all patients who had skin changes according to recommendations for skin biopsy use in clinical trials (6). In non-sclerotic GVHD, a 4-mm punch biopsy penetrating the subcutaneous adipose tissue was performed. In patients with sclerotic GVHD, a scalpel biopsy reaching the deep subcutis and, if necessary the fascia, was performed. Each patient was examined for skin tumors and pigmented lesions (nevi). Subjective symptoms, including pruritus, pain, and edema, were also evaluated.

### Statistical analysis

According to the results of Kolmogorov-Smirnov test and the small number of participants, appropriate non-parametric tests were used in all analyses. Categorical variables are shown as frequencies and corresponding percentages, and quantitative variables as medians and ranges. Fisher exact test or Fisher-Freeman-Halton exact test of independence when the contingency table was larger than 2 × 2 was used to analyze differences in categorical clinical parameters between patients (GVHD patients with alopecia areata and/or vitiligo) and controls (GVHD patients without alopecia areata and/or vitiligo) and Mann-Whitney U test was used to analyze the differences in quantitative variables. Binary logistic regression was performed to assess the impact of a number of factors on the likelihood that patients will have alopecia and/or vitiligo. The model contained four independent variables derived from previously made bivariate analyses and clinical relevance. *P* values below 0.05 were considered significant. Data analysis software system IBM SPSS Statistics, version 21.0 (*www.spss.com*, license grantee University of Zagreb, Faculty of Humanities and Social Sciences) was used in statistical analyses and graphical images.

## Results

### Patient characteristics

50 patients, 22 (44%) female, with a median age of 40 (range 9-71) who underwent allo-HSCT and were diagnosed with cGVHD by the NIH Consensus criteria (2) were enrolled into the study and were referred to the Dermatology Department for evaluation. Main indications for transplant were acute leukemia and myelodysplastic syndrome (62%) and myeloproliferative neoplasm (22%). 31 (62%) patients received myeloablative conditioning and 3 patients (6%) underwent total body irradiation (TBI) (Table 1). 31 patients (62%) received transplant from a related donor, 29 (58%) from a female donor, and 31 (62%) patients received peripheral blood stem cell as a cell source. 36 patients (72%) had previous acute GVHD of the skin. 23 (46%) presented with quiescent, 14 (28%) with *de novo,* and 13 (26%) with progressive onset, and 45 patients (90%) were classified as classic cGVHD. The majority of the patients (50%) were receiving no immunosuppression or mild intensity immunosuppression (8), and 52% had active GVHD by clinical impression defined as inactive off systemic therapy or topical immunosuppression, inactive on systemic therapy or topical immunosuppression, active irrespective of the level of current therapy and highly active irrespective of the level of current therapy. 3 patients (6%) had mild global NIH score, 20 (40%) moderate, and 27 (54%) severe global NIH score (Table 2).

**Table 1:** Demographic characteristics of the study population*

Characteristic	N (%)
Age	
median (range)	40 (9-71)
Patient sex	
female	22 (44)
male	28 (56)
Sex of the donor	
female	29 (58)
male	18 (36)
unknown	3 (6)
Diagnosis	
AML, ALL, MDS	31 (62)
CML, MPN	11 (22)
AA, PNH	5 (10)
NHL, HL, CLL	2 (4)
immunodeficiency	1 (2)
Stem cell source	
bone marrow	19 (38)
PBSC	31 (62)
Donor relationship	
related	31 (62)
unrelated	19 (38)
Conditioning regimen	
myeloablative conditioning	19 (38)
reduced intensity conditioning	31 (62)
Days from transplant to cGVHD diagnosis	
median (range)	288 (54-3886)
Days from cGVHD diagnosis to study enrolment	
median (range)	154 (0-7125)

^*AML – acute myeloid leukemia, ALL – acute lymphoblastic leukemia, MDS – myelodysplastic syndrome, CML – chronic myeloid leukemia, MPN – myeloproliferative neoplasm, AA – aplastic anemia, PNH – paroxysmal nocturnal hemoglobinuria, NHL – Non Hodgkin lymphoma, HL – Hodgkin lymphoma, CLL – chronic lymphocytic leukemia, PBSC – peripheral blood stem cell.^

**Table 2:** Transplant characteristics of in patients with graft-vs-host disease (GVHD) included in the study

Characteristic	N (%)
Acute GVHD	
yes	36 (72)
no	14 (28)
Acute skin GVHD	
yes	22 (44)
no	28 (56)
Chronic GVHD onset	
*de novo*	14 (28)
progressive	13 (26)
quiescent	23 (46)
Chronic cGVHD classification	
classic	45 (90)
overlap	5 (10)
Global National Institutes of Health Score	
mild	3 (6)
moderate	20 (40)
severe	27 (54)
Lines of prior systemic treatment	
median (range)	2 (0-6)
Intensity of immunosuppression	
none/mild	25 (50)
moderate	18 (36)
high	7 (14)
Chronic GVHD activity	
active	26 (52)
inactive	24 (48)

### Dermatological findings

Among 50 patients diagnosed with cGVHD, 28 (56%) had skin involvement. 11 patients had mild cutaneous NIH cGVHD score (39%), 6 had moderate (22%), and 11 patients (39%) had severe score. 27 (96%) patients had hypo and/or hyperpigmentation changes. 36 patients (72%) had a history of acute GVHD, all of whom had skin involvement at the time of diagnosis. In 25 of 28 (89%) patients with clinical diagnostic criteria for cutaneous cGVHD, skin biopsy was performed and histological diagnosis of cGVHD was established. In 3 of 28 patients skin biopsy was not performed because the patients refused the biopsy. 10 patients (20%) were diagnosed with vitiligo or alopecia areata; 4 of them had vitiligo (8%) and 6 (12%) had alopecia areata. In the vitiligo/alopecia areata group 4 patients also had sclerotic cGVHD.

**Figure 1:**
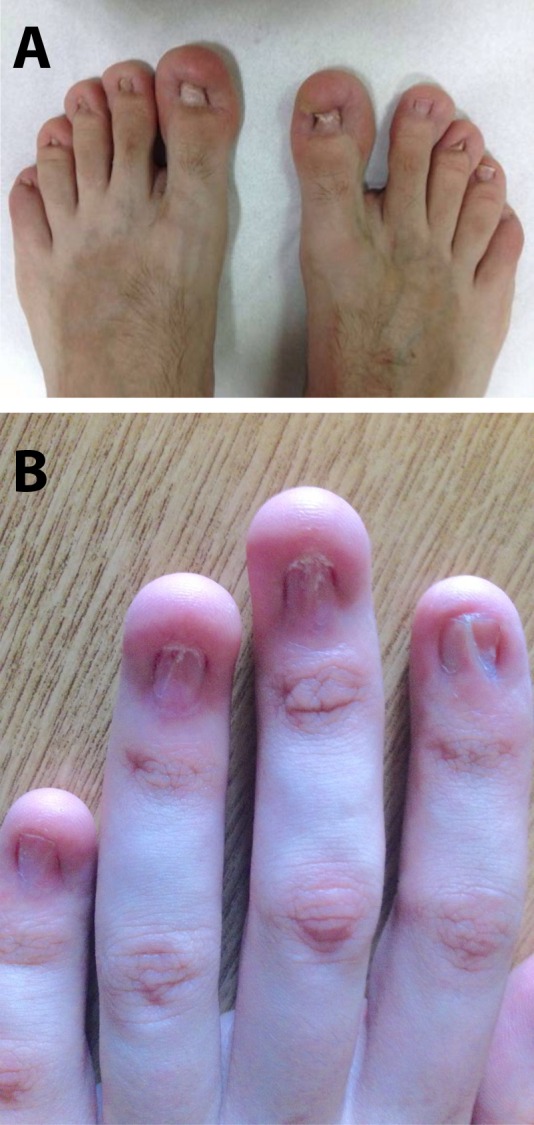
Pterygium in a patient with chronic graft-vs-host disease. (**A**) All fingernails and (**B**) all toenails. The image is published with the patient’s consent.

**Figure 2:**
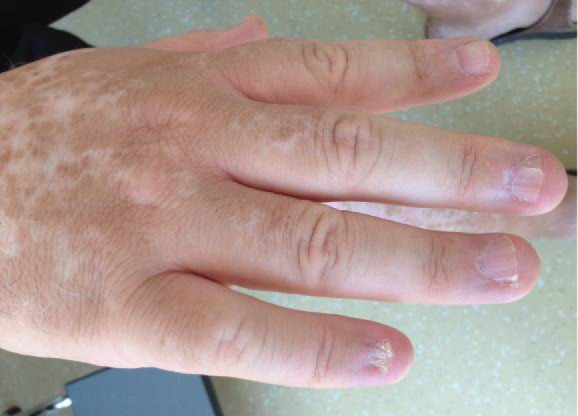
Pterygium and vitiligo in a patient with chronic graft-vs-host disease. The image is published with the patient’s consent.

Nail changes were observed in 15 (30%) patients, most commonly longitudinal ridging, splitting, or brittleness (12 patients). Pterygium unguis was observed in 2 patients, and onycholysis in 1 patient. In 1 male patient who had ophiasis pattern alopecia areata, pterygium was present on all the fingernails (Figure 1A) and toenails (Figure 1B), and in 1 male patient who had vitiligo, it was present on a single fingernail (Figure 2). Ulcers and erosions were observed in 6 patients, all of them were scored as severe cGVHD. Pruritus was observed in 15 (30%) patients and skin pain in 4 (8%) patients. 35 patients (70%) had skin xerosis.

As systemic immunosuppressive therapy is associated with an increased risk of cutaneous neoplasia, all patients underwent evaluation for cutaneous neoplasia and nevi. We did not find any malignant cutaneous neoplasia and we diagnosed 3 atypical nevi; they were sent for a total excision, and the diagnosis was pathohistologically confirmed as dysplastic nevi.

### Vitiligo and alopecia areata

**Figure 3:**
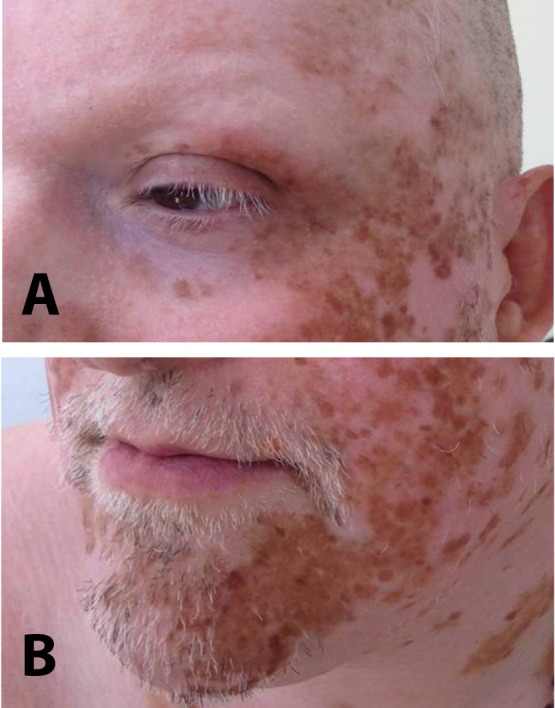
Vitiligo and leukotrichya in a patient with chronic graft-vs-host disease. (**A**) Eyebrow and eyelash; (**B**) beard. The image is published with the patient’s consent.

4 patients were diagnosed with vitiligo and all of them had the onset after the allo-HSCT. All patients with vitiligo were men, 2 of them had female donors (sisters), 1 had his brother as donor, and 1 had an unrelated HLA-matched donor (male). All vitiligo patients fulfilled clinical diagnostic criteria for cutaneous GVHD, and were histologically diagnosed as “consistent with GVHD” or “definitive GVHD” (1 like sclerotic type cGVHD and 3 like lichenoid cGVHD). 2 patients with vitiligo also had leukotrichia, 1 with completely white eyebrows and white eyelashes and the other with white left eyelashes (Figure 3A), and patches of white hair in the beard (Figure 3B). In addition, 2 vitiligo patients also complained of photosensitivity.

**Figure 4:**
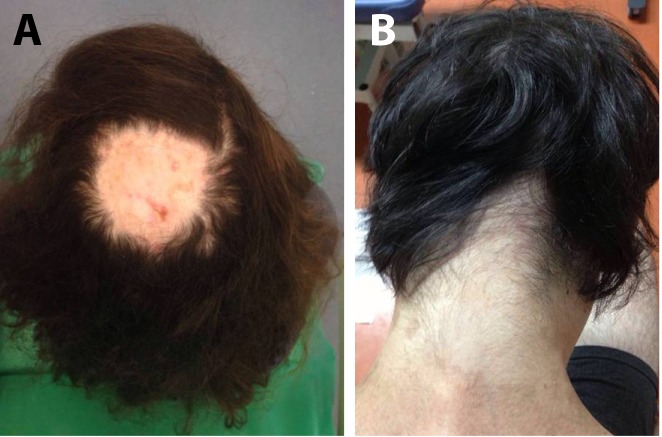
Alopecia areata in a patient with chronic graft-vs-host disease. (**A**) Frontoparietal; (**B**) ophiasis. The image is published with the patient’s consent.

**Figure 5:**
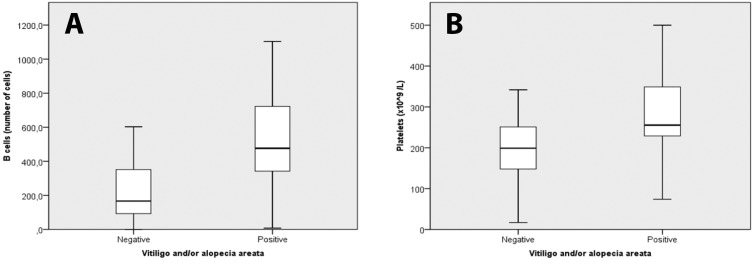
Vitiligo-generalized distribution in a patient with chronic graft-vs-host disease. (**A**) Back; (**B**) legs. The image is published with the patient’s consent.

11 patients had well developed changes in hair growth and in the rest of the body hair. Alopecia areata was diagnosed in 6 patients: 5 in the scalp (2 female children (Figure 4A), 2 adult women and 1 adult man), and in 1 male patient, in the beard. In another male patient, the beard did not grow at all. 1 male patient had ophiasis pattern of alopecia areata (Figure 4B). The other 4 patients had very thin hair. 1 female patient had lost both of her eyebrows and her pubic hair, but the scalp hair grew well after the transplantation. 2 patients with alopecia areata received TBI as part of conditioning. In the group of patients with alopecia areata, well-defined areas of round hair loss without scarring were distinguished from active skin GVHD at the site, persistent alopecia after recovery from chemotherapy and radiotherapy, scalp infections, telogen and androgen effluvium, and androgenetic alopecia. Alopecia areata was distinguished from other causes of alopecia on the basis of medical history, by clinical and dermoscopic examination and microscopy (culture). 1 patient had a generalized distribution of vitiligo (Figure 5A and 5B).

### Factors associated with vitiligo/alopecia areata

**Figure 6:**
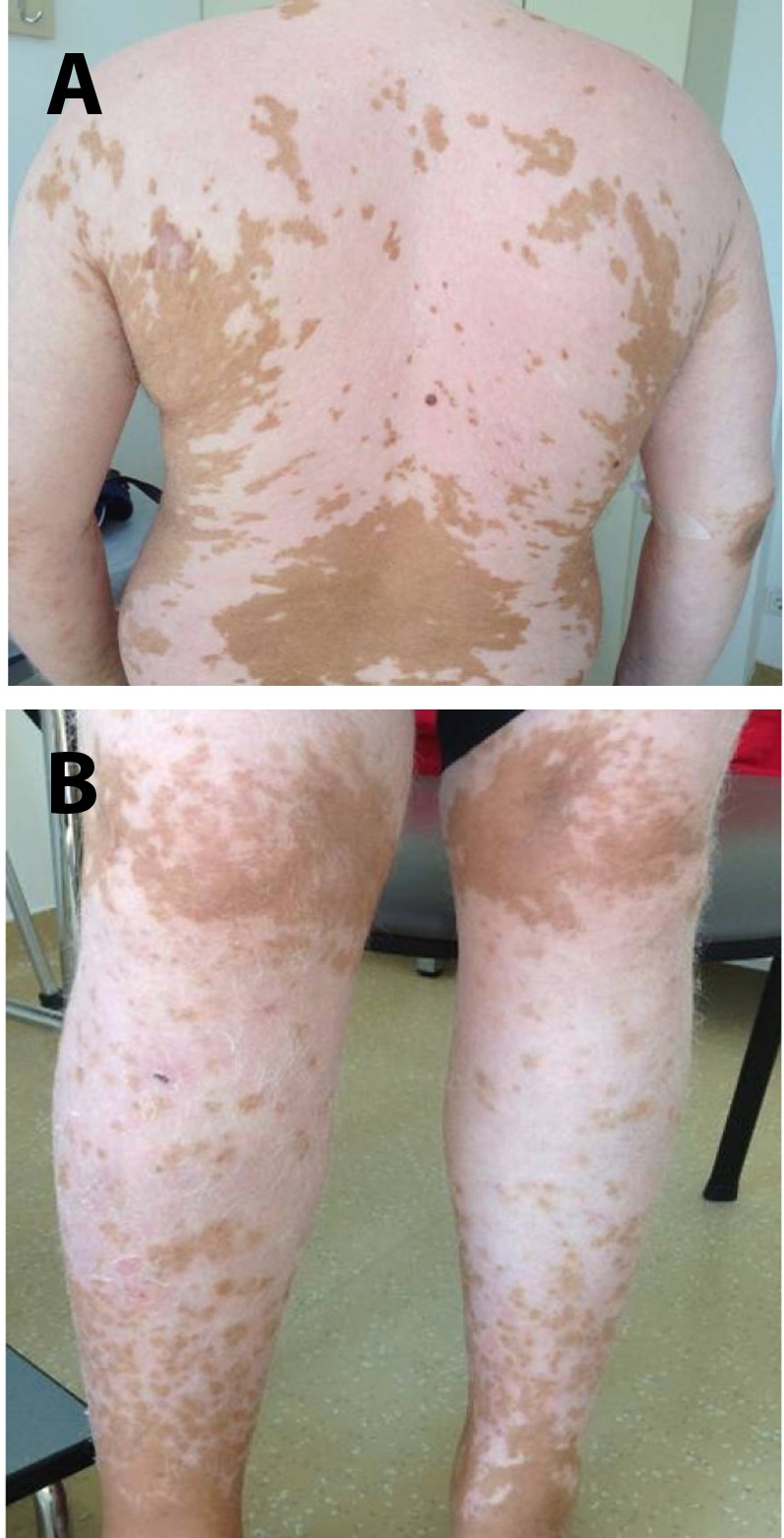
Univariate analysis of vitiligo/alopecia areata group of patients with chronic graft-vs-host disease. (**A**) B cells; (**B**) platelets.

Univariate analysis showed that patients with vitiligo/alopecia areata were younger (29 vs 44 years old, *P* = 0.028), received more lines of systemic immunosuppressive therapy (*P* = 0.043), had lower Karnofsky performance status (*P* = 0.028), had higher number of B-cells (*P* = 0.005), higher platelet count (*P* = 0.022), and higher total proteins (*P* = 0.024) than GVHD patients without alopecia areata and/or vitiligo. Vitiligo and alopecia areata were associated with higher NIH skin score (*P* = 0.001), higher intensity of immunosuppressive treatment (*P* = 0.020), and TBI conditioning (*P* = 0.040) (Table 3, Table 4, Figure 6). We did not find any differences between patients with and without vitiligo/alopecia areata in terms of sex of the donor, transplant cell source, prior acute GVHD, cGVHD onset, cGVHD classification, or organ involvement (except skin), global NIH score, and the majority of laboratory data (C reactive protein, albumins, complement components C3 and C4, etc), including antibodies (anticardiolipin IgG, anticardiolipin IgM, rheumatism factor).

**Table 3:** Univariate analysis of variables associated with vitiligo or alopecia areata in patients with graft-vs-host disease (GVHD) included in the study

	Vitiligo or alopecia areata						*P*
	Negative (N = 38)			Positive (N = 10)			
	25th	50th (median)	75th	25th	50th (median)	75th	
**Age at entry (years)**	28.00	44.00	53.00	18.00	29.00	36.00	0.028
**Days from chronic GVHD diagnosis to enrollment**	14.00	124.00	219.00	262.00	1175.50	3,184.00	0.002
**Lines of prior systemic therapy**	0.00	1.00	2.00	1.00	3.00	4.00	0.043
**Karnofsky score (%)**	80.00	90.00 100.00	70.00	80.00	90.00	0.028	
**B-cells (number)**	84.00	167.00	361.00	312.00	553.00	886.00	0.005
**Platelets ( × 10^9^g/L)**	148.00	199.00	251.00	222.00	255.50	368.00	0.022
**Total proteins (g/L)**	59.00	67.00	72.00	71.00	73.50	78.00	0.024

**Table 4:** Univariate analysis of variables associated with vitiligo or alopecia areata in patients with graft-vs-host disease (GVHD) included in the study

		Vitiligo or alopecia areata				*P*
		negative		positive		
		N	%	N	%	
Total body irradiation **conditioning**	no	34	100.00	7	77.80	0.040
	yes	0	0.00	2	22.20	
**Lines of prior systemic therapy**	0	12	32.40	2	22.20	0.003
	1	7	18.90	1	11.10	
	2	11	29.70	0	0.00	
	3	6	16.20	2	22.20	
	4	0	0.00	3	33.30	
	5	0	0.00	1	11.10	
	6	1	2.70	0	0.00	
**Intensity of immunosuppression (Sandy's scale)**	none/mild	17	44.70	7	70.00	0.020
	moderate	18	47.40	0	0.00	
	high	3	7.90	3	30.00	
**Disease activity**	inactive	17	44.70	8	80.00	0.075
	active	21	55.30	2	20.00	
**Skin National Institutes of Health (NIH) score**	0	21	55.30	1	10.00	0.001
	1	10	26.30	1	10.00	
	2	4	10.50	2	20.00	
	3	3	7.90	6	60.00	
**Global NIH score**	mild	21	55.30	1	10.00	0.442
	moderate	10	26.30	1	10.00	
	severe	4	10.50	2	20.00	

### Predictive factors for vitiligo/alopecia areata

Binary logistic regression model for prediction of vitiligo and alopecia areata included 4 independent variables (NIH skin score, age at study entry, number of B-cells, platelet count and global NIH score) derived from bivariate analyses (Table 5). The full model containing all predictors was statistically significant, X^2^ (5, N = 45) = 25.45, *P* < 0.001, and explained 66.1% (Nagelkerke R^2^) of dependent variable variance, and correctly classified 93.3% of the cases, indicating that the model was able to distinguish between GVHD patients who had from those who did not have alopecia and/or vitiligo. Hosmer-Lemeshow goodness of fit test result for this logistic regression model was non-significant (X^2^ = 7.75, *P* = 0.355), indicating that this regression model is applicable. NIH skin scoring was a significant predictive factor of alopecia and/or vitiligo – for each level of NIH skin scoring elevation patients were 3.67 times more likely to have alopecia and/or vitiligo (odds ratio 3.67; 95% confidence interval 1.26-10.73), controlled for all other factors in the model.

**Table 5:** Multivariate model for prediction of vitiligo or alopecia areata in patients with graft-vs-host disease (GVHD) included in the study

	Odds ratio (OR)	95% confidence interval		*P*
		lower	upper	
Age at entry	0.94	0.83	1.07	0.377
National Institutes of Health (NIH) skin scoring	3.68	1.26	10.73	0.017
B cells (number of cells)	1.00	1.00	1.01	0.091
Platelets ×10^9^ L	1.00	0.98	1.01	0.847
Global NIH score	2.38	0.20	28.49	0.495

## Discussion

We found a higher percentage of vitiligo and alopecia areata patients than expected compared to a prior retrospective report (9). Patients suffering from vitiligo and alopecia areata had higher NIH skin scores and clinical markers of immune activation, and more frequent and more severe nail changes than other cGVHD patients.

Vitiligo and/or alopecia areata are still considered uncommon phenomena in allo-HSCT recipients, although connection of vitiligo and cGVHD has already been established (7,9-16). Alajlan et al (7) described a patient with multiple myeloma in whom generalized vitiligo developed within 3 months after allo-BMT from his HLA-matched sister with vitiligo, and they proposed that adoptive transfer of donor immunity to the recipient may play a role in the development of vitiligo. In our study, not a single donor had vitiligo, and no one in the patients’ closer family suffered from vitiligo. Although other skin diseases, for example, psoriasis and allergic diathesis (eg, atopy), have also been described as being acquired by BMT recipients (17-19), we found no similar cases in our study.

Zuo et al (9) found that the risk factors for vitiligo and alopecia areata were female donor to male recipient sex mismatch and a positive test for cardiolipin antibody, and they identified 4.9% participants with vitiligo and 0.7% with alopecia areata (9). In our study, there was a much higher percentage of both vitiligo (8%) and alopecia areata (12%). We also did not find an association between vitiligo/alopecia areata and female donors. Another study found that patients with vitiligo/alopecia areata had higher B-cell count, perhaps consistent with cGVHD–related autoimmunity (20). However, we did not find an association with the presence of antibodies (anticardiolipin IgG, anticardiolipin IgM, rheumatism factor). Also, in our study vitiligo and alopecia areata were associated with higher platelet count, which in previous studies was associated with more active and more severe disease (21) and sclerotic cGVHD (22).

Our patients manifested a variety of vitiligo presentations, the most frequent among which were the classic periorbital, perioral, and acrofacial distributions, similar to the findings of Zuo et al (9). The majority of patients with hair changes also had nail involvement, which is well-described in patients with alopecia areata, but a link between ophiasis and pterygium has not been documented.

Although TBI has been associated with elevated risk of aGVHD and cutaneous cGVHD, to our knowledge, this is the first study in which an association between TBI and later development of vitiligo/alopecia areata has been found. However, we should interpret this result with caution, since there were only 2 patients who received TBI.

This study limitations are the small number of patients (n = 50) and the small total number of patients with specific dermatological manifestation. Despite the small number of patients, we think that the data presented here provide sufficient evidence that vitiligo and alopecia areata occur more frequently than has been published, and are associated with higher cGVHD severity and activity. Also, for the first time the Croatian patient population (an European cohort) with cGVHD was carefully monitored for dermatological manifestations. Considering the increasing incidence of cGVHD and the renewed research focus on this topic (23,24), further studies are needed to determine the true prevalence of vitiligo and alopecia areata in cGVHD patients and help find a better treatment for these patients, because they are frequently psychologically and cosmetically disturbed by these skin manifestations.
